# Ecto-nucleoside triphosphate diphosphohydrolase 3 in the ventral and lateral hypothalamic area of female rats: morphological characterization and functional implications

**DOI:** 10.1186/1477-7827-7-31

**Published:** 2009-04-22

**Authors:** David S Kiss, Attila Zsarnovszky, Krisztina Horvath, Andrea Gyorffy, Tibor Bartha, Diana Hazai, Peter Sotonyi, Virag Somogyi, Laszlo V Frenyo, Sabrina Diano

**Affiliations:** 1 Department of Physiology & Biochemistry, Szent Istvan University Faculty of Veterinary Science, Budapest, Hungary; 2 Department of Obstetrics, Gynecology & Reproductive Sciences, Yale University School of Medicine, New Haven, CT 06510, USA; 3 Department of Anatomy & Histology, Szent Istvan University Faculty of Veterinary Science, Budapest, Hungary; 4 Department of Neurobiology, Yale University School of Medicine, New Haven, CT 06510, USA

## Abstract

**Background:**

Based on its distribution in the brain, ecto-nucleoside triphosphate diphosphohydrolase 3 (NTPDase3) may play a role in the hypothalamic regulation of homeostatic systems, including feeding, sleep-wake behavior and reproduction. To further characterize the morphological attributes of NTPDase3-immunoreactive (IR) hypothalamic structures in the rat brain, here we investigated: 1.) The cellular and subcellular localization of NTPDase3; 2.) The effects of 17β-estradiol on the expression level of hypothalamic NTPDase3; and 3.) The effects of NTPDase inhibition in hypothalamic synaptosomal preparations.

**Methods:**

Combined light- and electron microscopic analyses were carried out to characterize the cellular and subcellular localization of NTPDase3-immunoreactivity. The effects of estrogen on hypothalamic NTPDase3 expression was studied by western blot technique. Finally, the effects of NTPDase inhibition on mitochondrial respiration were investigated using a Clark-type oxygen electrode.

**Results:**

Combined light- and electron microscopic analysis of immunostained hypothalamic slices revealed that NTPDase3-IR is linked to ribosomes and mitochondria, is predominantly present in excitatory axon terminals and in distinct segments of the perikaryal plasma membrane. Immunohistochemical labeling of NTPDase3 and glutamic acid decarboxylase (GAD) indicated that γ-amino-butyric-acid- (GABA) ergic hypothalamic neurons do not express NTPDase3, further suggesting that in the hypothalamus, NTPDase3 is predominantly present in excitatory neurons. We also investigated whether estrogen influences the expression level of NTPDase3 in the ventrobasal and lateral hypothalamus. A single subcutaneous injection of estrogen differentially increased NTPDase3 expression in the medial and lateral parts of the hypothalamus, indicating that this enzyme likely plays region-specific roles in estrogen-dependent hypothalamic regulatory mechanisms. Determination of mitochondrial respiration rates with and without the inhibition of NTPDases confirmed the presence of NTPDases, including NTPDase3 in neuronal mitochondria and showed that blockade of mitochondrial NTPDase functions decreases state 3 mitochondrial respiration rate and total mitochondrial respiratory capacity.

**Conclusion:**

Altogether, these results suggest the possibility that NTPDases, among them NTPDase3, may play an estrogen-dependent modulatory role in the regulation of intracellular availability of ATP needed for excitatory neuronal functions including neurotransmission.

## Background

Purinergic intercellular signaling has received much attention during the past decade. It has been known for some time that nucleotide-triphosphates, such as adenosine triphosphate (ATP), are not only energy carriers: ATP, for example, is a substrate for the production of its hydrolytic derivatives (ADP, AMP and adenosine) that are the specific ligands of different purinergic receptors (e.g., P_2_X, P_2_Y, P_1_) [1]. In fact, recent data suggest that purinergic signaling might be one of the first biological signaling systems that evolved during the phylogenesis [2]. The specific ligands of the relatively wide array of purinergic receptors are provided by the ATP-hydrolyzing activity of transmembrane ectonucleotidase enzymes (NTPDases) and 5'-ectonucleotidase. Of the known ectonucleotidases, NTPDase1-3 have been identified in the rat brain. NTPDase1 is widely expressed in neurons, glia and endothelial cells [3], while NTPDase2 was mainly found in the germinal zones of the rat brain, and is thought to play a role in neural development and differentiation [4]. NTPDase3 was cloned in 1998 by Smith and Kirley [5]; In 1998, Chadwick and Frischauf [6] demonstrated that NTPDase3 mRNA is most abundant in the brain and pancreas. The first description of the localization and distribution of NTPDase3 in the rat brain [7] has been recently published. In the latter study, NTPDase3 immunoreactivity (NTPDase3-IR) was only found in neuronal structures. The vast majority of IR profiles were axon-like neuronal processes concentrated in midline brain regions, with highest frequency in the hypothalamus, thalamus and the midbrain. Immunoreactive neuronal perikarya were only found in the lateral hypothalamic nucleus (LHN) and arcuate nucleus (AN). Based on those results, it has been suggested that, because of the high degree of region-specific distribution of immunoreactive profiles, NTPDase3 may play a role in one or more of the regulatory mechanisms of food-intake, sleep-wake behavior and reproductive physiology. While that previous light microscopic mapping of NTPDase3-IR in the rat brain provided useful information for further studies on purinergic signaling, understanding the cellular role of this enzyme warranted further determination of its subcellular localization and function. Therefore, here we characterized the intracellular localization of NTPDase3 in the hypothalamus of adult male rats. Electron microscopic results indicated the presence of NTPDase3-IR in neuronal perikarya and excitatory nerve terminals, but not in other (glial, vascular) cell types. To determine whether NTPDase3 is differentially or ubiquitously expressed in excitatory and/or inhibitory neuronal structures, we examined the possible co-localization of NTPDase3 with glutamic acid decarboxylase (GAD, the rate-limiting enzyme of the inhibitory neurotransmitter GABA) by means of immunohistochemistry. Considering that the ventrobasal hypothalamus is highly estrogen responsive, we also tested whether or not 17β-estradiol (E_2_) influences the expression level of NTPDase3 in hypothalamic tissue homogenates obtained from ovariectomized and ovariectomized plus E_2_-treated female rats. Finally, morphological indications of the presence of NTPDase3 in neuronal mitochondria implied a functional role for this enzyme in mitochondrial energy (ATP) production. Therefore, we also examined the effects of NTPDase inhibition on mitochondrial respiration in hypothalamic synaptosomal preparations.

## Methods

Sprague-Dawley male or female (as indicated below) rats were used for each study. Following the guidelines laid down by the NIH, the use of animals was approved by the respective University Committees on Animal Use at Yale University and Szent Istvan University Faculty of Veterinary Sciences.

### Animals, tissue fixation and immunolabeling

Male and female Sprague-Dawley rats (body weight: 230–250 g; vendor: Charles-River Laboratories, Inc.) were used. Animals were kept under standard laboratory conditions, with tap water and regular rat chow ad libitum in a 12-h light, 12-h dark cycle. For histological studies, brains of anesthetized (intramuscular injection of a mixture of 200 mg/kg ketamine and 6.6 mg/kg xylazine) ovariectomized (ovx) animals (n = 12) were fixed by transcardial perfusion of a mixture of 5% paraformaldehyde and 2% glutaraldehyde in 0.1 molar phosphate buffer and stored in 4% paraformaldehyde until tissue processing. For the electron microscopic analysis of subcellular localization of NTPDase3-IR, hypothalami were sectioned and 50 μm thick slices were immunostained for NTPDase3 using an affinity purified rabbit anti-NTPDase3 primary antibody. Omission of the primary antibody resulted in no detectable staining. (The rabbit anti-NTPDase3 [KLH14] primary antibody was kindly provided by Dr. Terence Kirley. Testing the specificity of this polyclonal antibody was described in details by Belcher et al. [7]). Immunostained sections were then processed (osmicated, dehydrated, embedded, sectioned for electron microscopy and contrast stained) for electron microscopic analysis as described in an earlier study [8]. In order to eliminate potential pitfalls arising from the possible precipitation of lead citrate, in addition to the general protocols we also examined ultra-thin sections that were only contrast-stained with uranyl-acetate during the dehydration process (1% uranyl-acetate in 70% ethanol for 60 minutes), but omitting subsequent treatment with lead citrate. For morphological characterization of synapses we considered the guidelines provided by Colonnier [9] and Palay and Chan-Palay [10].

To study the possible expression of NTPDase3 in GABAergic inhibitory neurons, we assessed whether NTPDase3 and GAD are co-expressed in hypothalamic neurons. Adjacent hypothalamic slices were used for the comparison of GAD (rabbit anti-GAD primary antibody, dil.: 1:2000; Sigma-Aldrich Chemie GmbH, Switzerland) and NTPDase3 immunolabelings by the previously described "mirror technique" [11]. In short, adjacent sections were arranged in pairs and one section of each pair was immunostained for NTPDase3 as described above, whereas their counterparts were single immunolabeled for GAD. Immunolabeling for GAD followed the standard immunohistochemistry protocol referred to above with the addition of a negative control experiment when the primary antibody for GAD was omitted. Omission of the primary antibody resulted in no detectable staining. After the visualization of immunoreactive material by nickel-intensified diaminobenzidine reaction, pairs of sections were thoroughly rinsed in 0.1 molar phosphate buffer and mounted with their matching surfaces on the upper side. Sections were then dehydrated through increasing ethanol concentrations and coverslipped.

Focusing the microscope on the upper surface of each section, digital images were captured at various magnifications and corresponding areas were determined based on the pattern of vasculature and matching cells halves through the overlay of images using Adobe Photoshop v. 7.0 software. After the computer-assisted reconstruction of the histological view, GAD-IR neurons were counted and potential NTPDase3-labeling of the matching cell-halves was searched.

### Western blot studies

Female animals were used for these studies. Rats were ovariectomized (ovx) and kept under standard laboratory conditions (as indicated above) for seven days. One week after ovariectomy, control animals were sacrificed (and processed as described below), while the rest of the animals received a single subcutaneous injection of 17beta-estradiol (23 μg/100 g body weight; Sigma, water-soluble, cat. no. E4389). Estrogen-primed rats were then sacrificed 2–26 hours after receiving the estrogen in two-hour intervals to determine the temporal changes in blood estrogen concentrations and hypothalamic NTPDase3 expression (n = 5 for each group). After quick decapitation, a tissue block containing the AN and LHN was excised, following the coordinates of the rat brain atlas [12] as follows: A coronal slice of the entire rat brain was cut with a rostral border at anterioposterior level 2.12 mm behind the bregma (just behind the caudal border of the optic chiasm) and a caudal border at anterioposterior level 4.52 mm behind the bregma (just before the caudal tip of the mamillary body). Slices were divided into two halves along the midsagittal plane. The dorsal border of the tissue block was cut along a horizontal line dorsally tangential to the third ventricle and the remaining cortical tissue and optic tract were removed. The remaining tissue block was further divided into two halves along a sagittal plane passing through the fornix. Thus, we obtained two tissue blocks from the medial part of the hypothalamus containing the AN, and two from the lateral part containing the LHN from each animal. Tissue blocks were then homogenized in (in mM) 20 Tris-HCl, pH 7.5, 150 NaCl, 1 PMSF, 1 EGTA, 1 EDTA, 2.5 sodium pyrophosphate, 1-beta-glicerol phosphate, and 1 Na_3_VO_4 _plus 1 mg/ml Pefabloc, 10 μg/ml leupeptin 10 μg/ml pepstatin, 1 μg/ml aprotinin, and 1% Triton X-100, 0.05% sodium deoxycholate. Homogenates were sonicated for 5 sec a total of 5 times and cleared by centrifugation at 14,000 × g for 1 min at 2°C. Protein concentrations were determined with a BCA protein assay kit (Pierce, Rockford, IL). Western blotting and densitometric analysis were performed by standard protocols [13,14]. Membranes were blocked with 5% nonfat dry milk for 1 hr in TBS-T and incubated with appropriate antisera (affinity purified rabbit anti-NTPDase3, KLH14, as described by Belcher et al. [7]). Immunoreactive bands were visualized onto preflashed x-ray film by enhanced chemiluminescence. Multiple exposures of each blot were collected, and those in the linear range of the film were used for densitometric analysis. Optical densities were calculated as arbitrary units after local area background subtraction, normalized to the protein concentrations of samples and to the density of controls. Results are reported as fold changes relative to control. All data that have been presented are representative of at least three independent experiments.

Serum estradiol concentrations were determined from each animal used for the western blot studies by 3H-RIA, as described by Csernus [15].

### Brain synaptosomal preparation and measurement of oxygen consumption

Male animals (n = 8) were used for the determination of mitochondrial respiration rates. Animals were anesthetized with isofluorane and brains were removed after quick decapitation. Hipothalami were dissected and cut into two halves. One side was always used as control (incubated in vehicle), while the contralateral side was used to determine the effects of the NTPDase inhibitor suramin (Calbiochem, San Diego, CA). The ability of suramin to block the enzymatic function of NTPDases has been previously reported [16]. Samples were homogenized in isolation buffer (pH 7.2; 215 mM mannitol, 75 mM sucrose, 0.1% bovine serum albumin, 1 mM EGTA, 20 mM HEPES). The homogenate was spun at 1300 × *g *for 3 min, the supernatant was removed, and the pellet was resuspended with isolation buffer and spun again at 1300 × *g *for 3 min. The two sets of supernatants from each sample were topped off with isolation buffer and spun at 13,000 × *g *for 10 min. The supernatant was discarded, and the step was repeated. After this second spin at 13,000 × *g*, the supernatant was discarded, and the pellets were resuspended with isolation buffer without EGTA and spun at 10,000 × *g *for 10 min. The final synaptosomal pellet was resuspended with 50 μl of isolation buffer without EGTA [17,18]. Protein concentration of mitochondrial suspensions was determined with a BCA protein assay kit (Pierce, Rockford, IL). As mentioned above, one half of the samples collected (i.e., the right side of the hypothalamus) was treated with suramin at a final concentration of 20 μM followed by a 30 minutes incubation of all samples at 37°C just before the measurements. Control sides were incubated for 30 minutes at 37°C in vehicle. Mitochondrial respirations were assessed using a Clark-type oxygen electrode (Hansatech Instruments, Norfolk, UK) at 37° with pyruvate and malate (5 and 2.5 mM; State 2 respiration) as oxidative substrates in respiration buffer (215 mM mannitol, 75 mM sucrose, 0.1% fatty acid-free BSA, 20 mM HEPES, 2 mM MgCl, 2.5 mM KH_2_PO_4_, pH adjusted to 7.2 with KOH). For analysis of ADP dependent respiration (state 3 respiration), ADP was added after the addition of oxidative substrates. After the addition of oligomycin (state 4 respiration), mitochondrial respiration was measured as increased fatty acid-induced respiration (Palmitate 150 μM). Total uncoupled respiration was also measured after the addition of the protonophore FCCP (carbonylcyanide-4-(trifluoromethoxy)-phenylhydrazone, 1 μM). The results are expressed as nmols of oxygen consumed per minute per mg protein.

### Statistical analyses

Statistical analyses were conducted with a Student's t test or by one-way ANOVA with Tukey's Multiple Comparison Test as appropriate. Data were analyzed with Excel (Microsoft) and GraphPad Prism version 4 (GraphPad Software, San Diego, CA)

## Results

### Light- and electron microscopy

**Figure 1:**
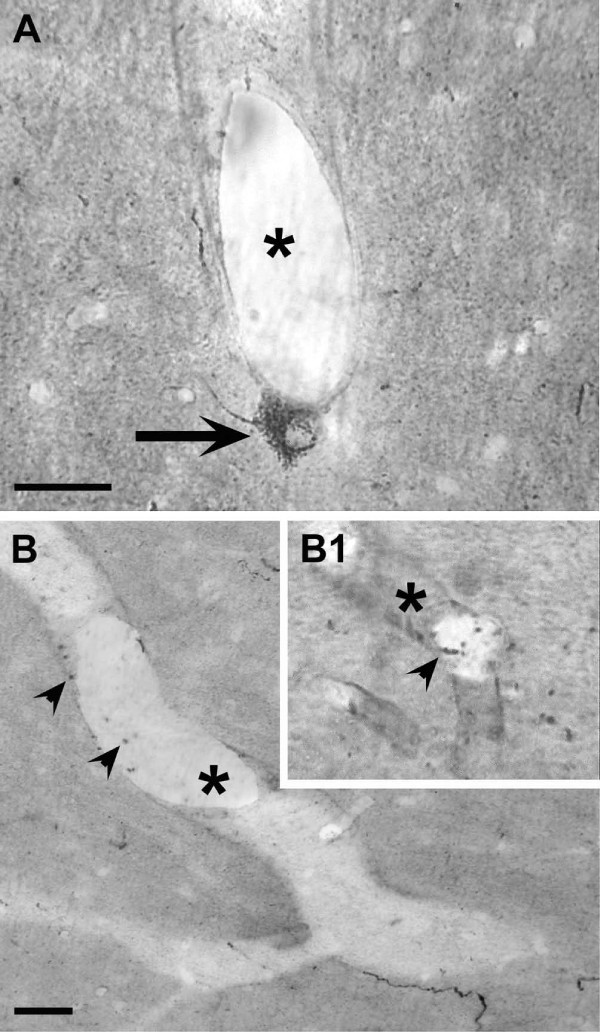
**Hypothalamic NTPDase3-immunoreactive cells in close apposition to hypothalamic vessels**. NTPDase3-immunoreactive perikarya (**A**, arrow) and putative neuronal processes (**B**, arrowheads) were frequently seen in close apposition to hypothalamic capillaries (asterisks). Scale bars represent 20 μm.

**Figure 2:**
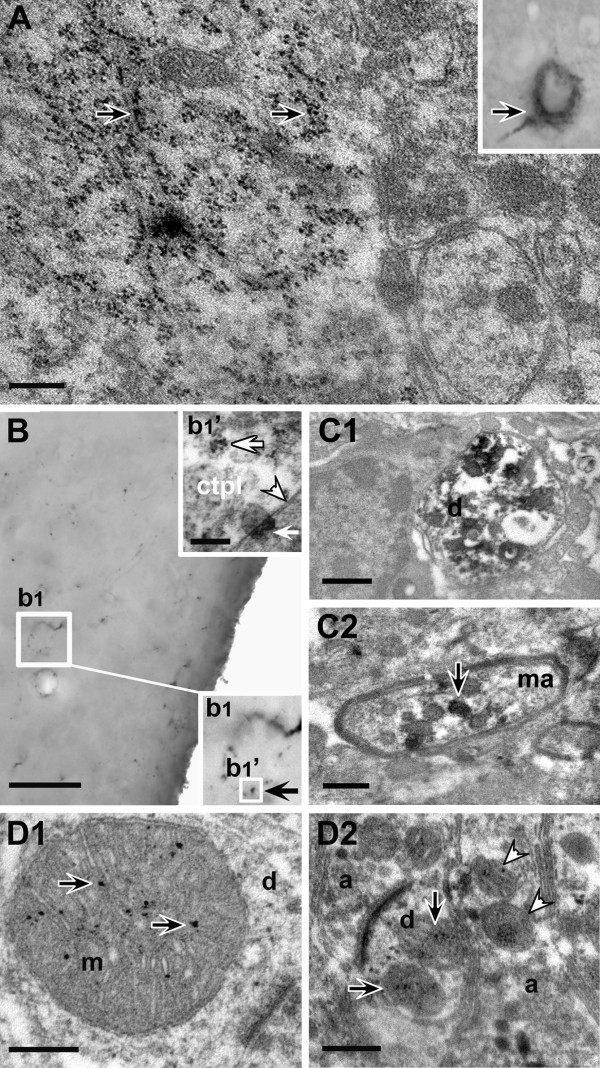
**Electron microscopic analysis of ecto-nucleoside triphosphate diphosphohydrolase 3-(NTPDase3) immunoreactive hypothalamic profiles**. **A**: Grainy cytoplasmic immunoreactivity (inset, black arrow) may result partly from NTPDase3-IR material linked to ribosomes (black arrows). Scale bar: 400 nm. **B**: Light microscopic studies showed NTPDase3-immunoreactive dots (**b1 **inset, black arrow) outlining the boundaries of immunoreactive cells. Scale: 50 μm. Correlated electron microscopic examination showed that immunoreactive dots shown on **b1 **represent plasma membrane-linked aggregates with Nickel-DAB-labeling on the extracellular side (**b1'; **Arrowhead points to the plasma membrane. Upper white arrow indicates cytoplasmic immunopositive material; Lower white arrow points to a plasma membrane-bound immunoreactive material. Scale bar: 100 nm.) ctpl: cytoplasm. **C1**: Representative image of an NTPDase3-immunoreactive dendrite (d). Scale bar: 0.5 μm. **C2**: Representative image of a myelinated axon (ma) containing NTPDase3-immunoreactive mitochondria (arrow). Scale bar represents 1 μm. **D1**: NTPDase3-immunoreactive material in the mitochondrial (m) matrix (arrows) or linked to the inner mitochondrial membrane in a dendrite (d) Scale: 200 nm. **D2**: NTPDase3-immunoreactive mitochondria in a dendritic (d) spine (black arrows) near an asymmetric synapse and in an unmyelinated axon (a) segment (white arrowheads). Scale: 400 nm.

The hypothalamic distribution of NTPDase3-IR found in the present study was consistent with that described in an earlier report [7]. Light microscopic analysis of IR profiles showed NTPDase3-IR cell bodies and neural-like processes in the LHN and AN, whereas in the rest of the hypothalamus only immunostained cell processes were found, many of which were morphologically closely associated with the vasculature (Figure 1). A more detailed examination revealed that cellular staining occurred either in the form of cytoplasmic staining predominantly aggregated in particle-like dots (Figure 2A) or as plasma membrane-associated punctate structures (Figure 2B). Correlated electron microscopic analysis of neuronal membranes showed that NTPDase3-IR is present at certain well-demarked segments of the plasma membrane (Figure 2Bb1).

Electron microscopic analysis clarified that labeled neuronal processes comprise both dendrites (Figure 2C1) and axons (Figure 2C2). In dendrites, cytosolic, ribosome-associated, as well as mitochondrial labelings were detected (Figure 2D1–D2). In contrast, in myelinated axons and axon terminals only mitochondrial immunoreactivity was observed. NTPDase3-IR was found near asymmetric (therefore considered putative excitatory), but not symmetric (inhibitory), synaptic membrane specializations (presynaptic axon terminals, dendrites and dendritic spines). The ultrastructural appearance of the grainy cytoplasmic- and dot-like or line-shaped membrane-associated immunoreactive material was also investigated in neuronal perikarya of the LHN and AN.

Ultrastructural examination of the cytoplasmic labeling revealed that part of the cytoplasmic IR particles observed in light microscope are associated with ribosomes, some of which are free cytoplasmic, but the majority of which are linked to the endoplasmic reticulum. Interestingly, we found NTPDase3-IR in the mitochondrial matrix or closely associated with the inner mitochondrial membrane (Figure 2D1–D2). Immunolabeled mitochondria were typically linked to asymmetric (putative excitatory) synaptic membrane specializations, present either in presynaptic terminals or at the post-synaptic side, in dendritic spines. Labeled mitochondria were also detected in the perikaryal cytoplasm, frequently concentrated in the vicinity of the plasma membrane.

### Immunolabeling for NTPDase3 and GAD

**Figure 3:**
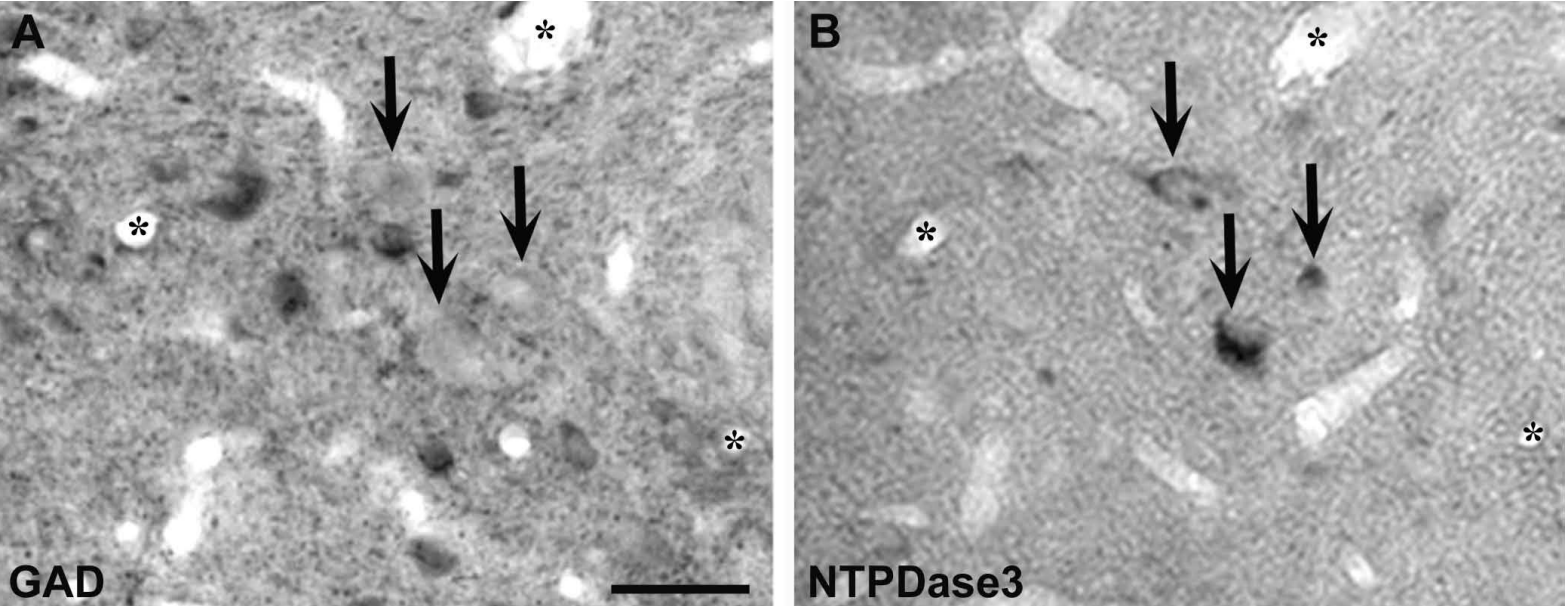
**Immunolabeling for glutamic acid decarboxylase (GAD) and ecto-nucleoside triphosphate diphosphohydrolase 3 (NTPDase3) immunoreactivities in the hypothalamus using the mirror technique**. Corresponding arrows point to matching cell halves immunostained either for GAD (**A**) or NTPDase3 (**B**). None of the examined GAD-immunoreactive neurons contained NTPDase3-immunoreactive material. Asterisks label corresponding vessels. Scale: 50 μm.

Of the 320 NTPDase3-IR cell bodies examined, 29 contained nuclei with invaginations of the nuclear membrane. Since GABAergic neurons of the AN possess infolded cell nuclei [19,20], we assessed whether NTPDase3 is expressed in GABAergic (and thus inhibitory) cells. We compared the hypothalamic localization of GAD and NTPDase3 (Figure 3). None of the 2540 GAD-IR neurons examined contained NTPDase3, indicating that NTPDase3 may be predominantly expressed in excitatory neurons of the hypothalamus.

### Western blot; Estrogen effects on hypothalamic NTPDase3 expression

**Figure 4:**
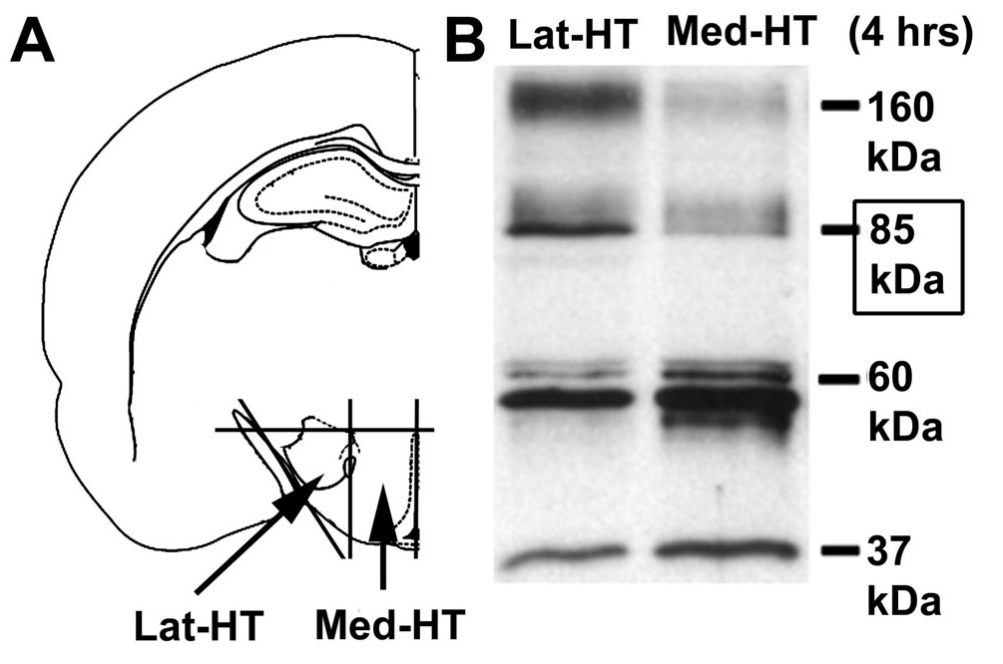
**Assessment of NTPDase3-levels in the hypothalamus of female rats**. **A**: Sample collection. Tissue blocks of the medial part of the hypothalamus (Med-HT) containing the arcuate nucleus and the lateral part of the hypothalamus (Lat-HT) containing the lateral hypothalamic nucleus were excised as follows: A coronal slice of the entire rat brain was cut with a rostral border at anterioposterior level 2.12 mm behind the bregma (just behind the caudal border of the optic chiasm) and a caudal border at anterioposterior level 4.52 mm behind the bregma (just before the caudal tip of the mamillary body). Slices were cut into two halves along the midsagittal plane. The dorsal border of the tissue block was cut along a horizontal line dorsally tangential to the third ventricle and the remaining cortical tissue and optic tract were removed. The remaining tissue block was further cut into two halves along a sagittal plane passing through the fornix. Thus, we obtained two tissue blocks from both the medial and lateral parts of the hypothalamus from each animal. **B**: Representative image showing NTPDase3-immunoreactive material in the Lat-HT and Med-HT four hours after a single subcutaneous injection of 17β-estradiol. Four immunoreactive bands were detected using homogenates of whole tissue samples. The 82–85 kDa molecular weight form of the enzyme, generally considered as the fully functional form, was further analyzed. Note the difference between the densities of the 82–85 kDa bands of the Lat-HT and Med-HT samples.

**Figure 5:**
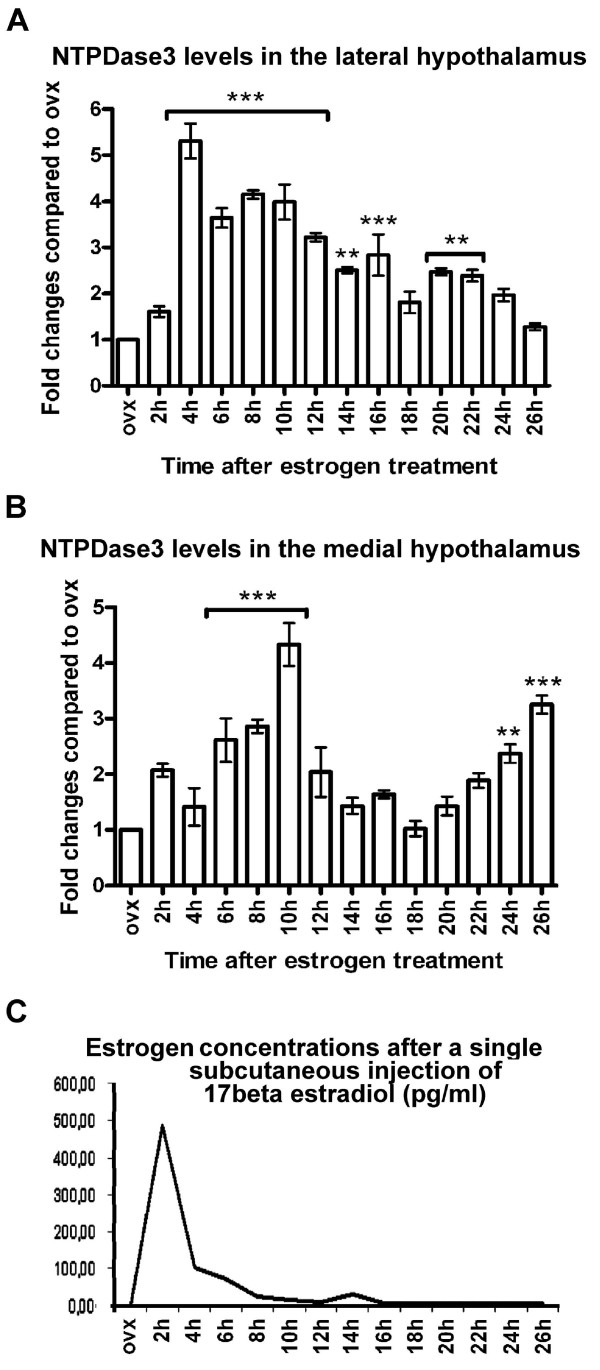
**Time-course of changes in hypothalamic NTPDase3-levels after a single subcutaneous injection of 17β-estradiol (E_2_)**. Optical density measurements of the 82–85 kDa immunoreactive bands were analyzed in ovx animals and ovx plus E_2_-treated animals 2–26 hours after a single subcutaneous injection of E_2_. Samples were taken in two-hours intervals after E_2_-priming. **A**: Estrogen-treatment induced a sharp rise in NTPDase3-expression in lateral hypothalamic samples, reaching significantly elevated peak levels by four hrs. After the peak, a gradual decrease in NTPDase3-expression was observed reaching nearly control ovx levels by 26 hrs. **B**: In medial hypothalamic samples, E_2_-treatment induced a gradual increase in NTPDase3-levels with highest values observed at 10 hrs after E_2_-priming. This was followed by falling of NTPDase3-level that reached a nadir at 18 hrs, followed by a second rise by 24–26 hrs. The difference in the time-course of changes in NTPDase3-levels between the medial and lateral part of the hypothalamus suggests that NTPDase3 may serve region-specific functions in the neuroendocrine hypothalamus. **C**: Mean E_2 _blood plasma concentrations measured after a single subcutaneous injection of E_2 _(water soluble, 23 μg/100 g body weight).

Since NTPDase3-IR perikarya were only detected in two of the hypothalamic regions, i.e., the AN and LHN, isolated medial and lateral hypothalamic tissue samples were collected from ovariectomized and estrogen-primed animals, and examined by western blot analysis for the expression level of NTPDase3 (Figure 4). Four immunoreactive bands (~160–170 kDa, 82–85 kDa, 60 kDa and 37 kDa) were detected using the rabbit anti-NTPDase3 affinity-purified polyclonal antibody (KLH14, kindly provided by Dr. Terence Kirley, University of Cincinnati, OH, USA). In a previous study, where this antibody was tested and used for western blot studies, multiple immunoreactive bands were found using COS cell membranes, and one IR band was detected using rat brain thalamus membranes from ovariectomized rats [7]. It is, therefore, not surprising that using homogenates of rat hypothalami (a brain area highly sensitive to estrogen) more IR bands were found, likely representing distinct structural and functional forms of the enzyme. Of the listed bands, the 85 kDa protein is considered as the fully glycosilated form of the protein, while the 160–170 kDa and 60 kDa values correspond to the dimeric form of the enzyme and the core protein. Here we focused on the analysis of the expression level of the 85 kDa protein. A time- and estrogen dependent increase in NTPDase3 levels was found in both (medial and lateral) selected hypothalamic areas (Figure 5); however, the temporal changes in NTPDase3 levels displayed distinct patterns. In samples including the LHN, NTPDase3 levels increased significantly 4–12 hrs after a single subcutaneous injection of E_2_, and gradually returned to nearly control (ovx) levels by 16–26 hrs after E_2 _treatment (Figure 5A). In contrast, temporal changes in medial hypothalamic samples including the AN showed an initial increase in NTPDase3 expression between 6–10 hrs after E_2 _treatment, followed by a sharp decrease to control level, and again followed by a second rise between 22–26 hrs after E_2 _treatment (Figure 5B). Thus, in the lateral hypothalamus a single-, whereas in the medial hypothalamus a double-peaked curve was determined, suggesting that NTPDase3 expression in the hypothalamus is regulated by E_2_, however, its role in the two hypothalamic regions might be different.

### Mitochondrial respiration measurements

**Figure 6:**
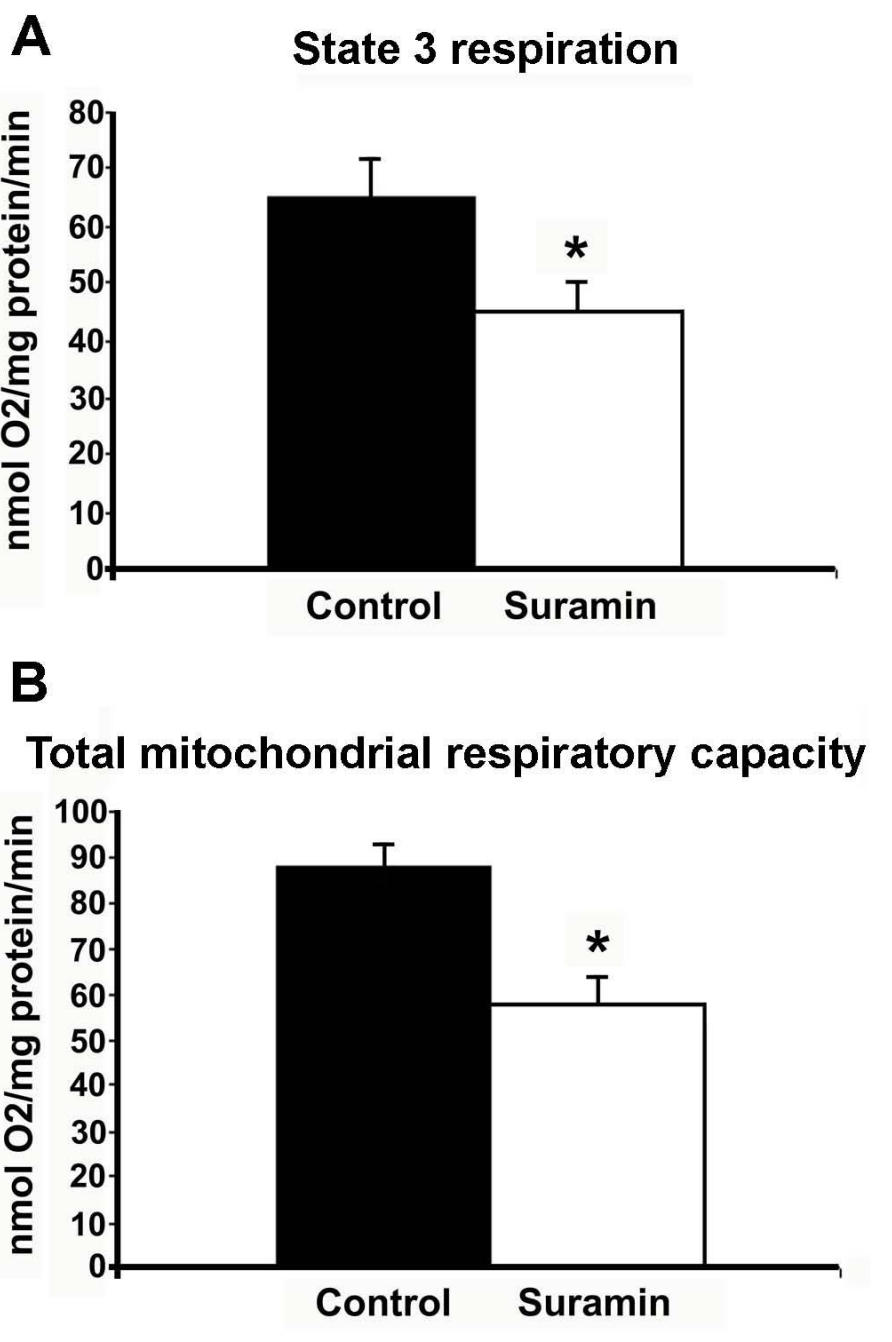
**Determination of mitochondrial respiration rates in hypothalamic synaptosomes**. Inhibition of ecto-nucleoside triphosphate diphosphohydrolase-activity by Suramin decreased oxygen consumption during state 3 mitochondrial respiration (**A**), and also decreased the total mitochondrial respiratory capacity (**B**).

NTPDase3 has been known as plasma membrane-incorporated ATP hydrolyzing enzyme with its active domain outside of the cell, thus converting ATP to ADP and AMP in the extracellular space [5]. According to current knowledge, the hydrolysis of P_2 _nucleotide agonists (nucleoside tri- and di-phosphates) to respective monophosphates by NTPDases is coupled to the hydrolysis of the nucleoside monophosphates by 5'-ectonucleotidase (also described as CD73) to generate adenosine, which can activate P_1 _adenosine receptors. Therefore, our finding that NTPDase3-IR is present in neuronal mitochondria was intriguing and warranted functional support of the morphological results. To study the functional link between NTPDase3 and mitochondria, we investigated the effect of the NTPDase inhibitor, suramin, on mitochondrial functions. It is important to note that to the best of our knowledge, there is no selective NTPDase (including NTPDase3) inhibitor available on the market. Muller et al. [16] compared and characterized the ability of polyoxomethalates (the best known NTPDase inhibitors) to block the functions of the different NTPDases (and purinergic receptors). Based on those results, we chose to use suramin, which was described as the most potent NTPDase3 inhibitor among the compounds examined, however, it also inhibits other NTPDases. Therefore, in the present study the effects of suramin treatments are considered as general inhibitory effects on NTPDases, including NTPDase3, until a fully selective NTPDase3 blocker becomes available. Inhibition of NTPDases by suramin had a significant inhibitory effect on State 3 mitochondrial respiration (Figure 6A) (45.05 ± 4.9 nmol O2/mg protein/min with suramin versus 65.1 ± 6.6 of control; P < 0.05) indicating that a decrease in ATP-ADP conversion occurred in mitochondria after NTPDase inhibition. All other respiration states were unaffected by the blockade of NTPDase function. However, the total mitochondrial respiratory capacity decreased as well (Figure 6B) (87.8 ± 5.5 nmol O2/mg protein/min in control synaptosomes versus 57.7 ± 6.5 nmol O2/mg protein/min (P < 0.05) in synaptosomes incubated with suramin), suggesting that mitochondrial NTPDase activity likely influences oxygen-consuming biochemical processes in the mitochondrial matrix.

## Discussion

### Light- and electron microscopy

An earlier mapping study [7], reported that in the rat brain, NTPDase3 is present exclusively in neurons, mostly in neuronal processes. In the present study, we confirmed those previous results, showing IR cell bodies localized in the LHN and AN, while in the rest of the hypothalamus only cell processes were observed, frequently in close apposition to the wall of hypothalamic vessels. The latter observations raise the possibility that at least part of NTPDase3-IR hypothalamic cells might be involved in the mediation of peripheral humoral signals by either the release of neuronal end-products into the blood circulation, and/or, intracellular events of such neurons could be affected by peripheral humoral factors through a yet unidentified NTPDase3-mediated intracellular signaling system.

Our light- and correlated electron microscopic studies showed that NTPDase3-IR is present at certain well-demarked segments of the plasma membrane. This finding is consonant with the generally accepted view of NTPDases being transmembrane proteins and hydrolyzing phosphorylated nucleotides outside of the cell. With respect to the cytoplasmic immunoreactivity, part of the cytoplasmic IR particles was associated with ribosomes, some of which were free cytoplasmic, but the majority of which were linked to the endoplasmic reticulum. This finding is not surprising in light of the fact that the amino acid sequence used for the generation of the antibody is part of the core protein; therefore, it is immunohistochemically detectable readily after translation. Another source of immunoreactive cytoplasmic particles appeared to be linked to mitochondria.

The detection of NTPDase3-IR in the mitochondrial matrix or closely associated with the inner mitochondrial membrane is probably the most intriguing finding of this study. Since immunolabeled mitochondria were typically found near asymmetric (putative excitatory) synaptic membrane specializations, it is reasonable to assume that mitochondrial NTPDase3 activity is functionally linked to excitatory, rather than inhibitory neuronal functions.

### Immunolabeling for NTPDase3 and GAD

NTPDase3-IR was found in the vicinity of asymmetric (therefore presumably excitatory), but not symmetric, synaptic membrane specializations (presynaptic axon terminals, dendrites and dendritic spines). Additionally, by immunolabeling for GAD and NTPDase3 and applying the well-known mirror-technique, we found that none of the GAD-IR neurons examined contained NTPDase3. Although we have not examined all hypothalamic synapses and GAD-IR neurons for the localization of NTPDase3, and some other, non-GABAergic neurons (e.g., dopaminergic) that are inhibitory in function may also express NTPDase3, our present findings still suggest that the vast majority of hypothalamic NTPDase3 is expressed in excitatory neurons.

In dendrites, free cytosolic (a possible soluble form), ribosome-associated, as well as mitochondrial labelings were detected. In contrast, in myelinated axons and axon terminals only mitochondrial immunoreactivity was observed. While the versatility in the appearance of immunoreactive material in dendrites may refer to the cellular processes of NTPDase3-metabolism (biosynthesis, protein maturation, explantation and organelle-linked function), the mitochondrial presence of this enzyme in axons and axon terminals suggests that the modulation of mitochondrial ATP-levels required to fuel neuronal output may be, at least to some extent, regulated by NTPDase3 activity.

### Estrogen effects on hypothalamic NTPDase3 expression

As mentioned earlier, multiple NTPDase3-IR bands were detected in western blot studies using rat hypothalamus homogenates. This phenomenon may indicate that there is "incomplete" processing of the enzyme, however, it is more likely that the distinct bands observed represent different maturational forms of the enzyme. In this study, we detected NTPDase3-IR linked to multiple subcellular structures (plasma membrane, ribosomes, endoplasmic reticulum) including neuronal mitochondria. Additionally, we provided evidence for NTPDase-activity in synaptosomal preparations. Therefore, it is also possible that one or more of the protein-forms detected on western blots represent functional forms of the protein adapted or adjusted to the microenvironment or functional attributes of the cell organelles.

Since the neuroendocrine hypothalamus is highly E_2_-responsive, it was reasonable to assume that E_2 _may influence the expression level of NTPDase3 within this brain area. Therefore, in a pilot study we investigated whether E_2_-treatment of ovx animals affects NTPDase3-levels in tissue blocks containing the entire hypothalamus. That study showed that a single subcutaneous injection of E_2 _results in significantly increased levels of NTPDase3 (unpublished observation). Those results prompted us to examine and analyze the temporal changes in NTPDase3-expression in lateral- and medial hypothalamic tissue samples as described above. The present findings indicate that in response to a single subcutaneous injection of E_2_, NTPDase3 expression increases in just a few hours after E_2_-treatment in both hypothalamic areas, however, the pattern of temporal changes in the medial hypothalamus differs from that observed in the lateral part of this brain area. Since the mediobasal hypothalamus, including the AN, is a major player in the biphasic (positive- and negative feedback) regulation of the gonadotrophin secretion and release, it is reasonable to speculate that in the medial part of the hypothalamus, NTPDase3 may be involved in the estrogenic control of gonadotrophins. The idea of a causal coincidence between the two peaks in NTPDase3 levels and the positive/negative gonadotrophin feedbacks raises several questions. For example, a number of data suggest that NTPDase3 may be involved in the hypothalamic regulation of gonadotrophin release. We have previously described that during the E_2_-induced gonadotrophin surge, an E_2_-dependent synaptic reorganization on hypothalamic neurons occurs. This phenomenon we termed "phased synaptic remodeling", that shows specific changes in the ratio of inhibitory/excitatory synapses during the two (positive- and negative-) states of the gonadotrophin feedback control [21]. A sharp rise in the number of excitatory synapses was observed at the time of the E_2_-surge, and the formation of new synapses may very well include ones equipped with NTPDase3-containing mitochondria. This hypothesis is consonant with our observation that inhibition of NTPDase activity decreases state 3 mitochondrial respiration and the total mitochondrial respiratory capacity, ergo an increased amount of mitochondrial NTPDase would well serve the energy-needs of a transient intensification in excitatory neuronal activity.

Both medial and lateral hypothalamic functions are known to involve mechanisms mediated by various purinoceptors, such as A_1_, P_2_X, and the activity of NTPDase3-containing hypocretin-orexin neurons in the LHN is directly influenced by such receptor actions [22-29]. Since here we found neuronal membrane-linked NTPDase3-IR in the AN/LHN, and E_2 _induced a transient increase in NTPDase3-levels in both hypothalamic sites, it is also possible that E_2 _increases the amount of membrane-incorporated NTPDase3 to transiently intensify purinergic interneuronal signaling. Further studies are underway to clarify this issue.

In lateral hypothalamic samples, NTPDase3 levels peaked at 4 hrs after E_2_-treatment followed by a gradual decrease, and reached ovx levels by 26 hrs. It has been demonstrated by Belcher et al. [7] that LHN NTPDase3-containing cells are nearly all (96–97%) hypocretin-orexin-containing neurons. These neurons are known to be direct modulators of the midbrain raphe serotonergic neurons [30] to influence sleep-wake states. It is also known that E_2 _influences arousal mechanisms in many ways [31]. Thus, it is possible that the mechanism through which E_2 _facilitates wakefulness involves increased NTPDase3-activity. On the other hand, LHN hypocretin-orexin (plus NTPDase3-IR) neurons are not only targets of E_2_, but also that of the gastric hormone ghrelin; at the same time, these neurons also represent the major excitatory input of AN neuropeptide Y/Agouti-related protein-containing cells whose activity is responsible for the initiation of food intake. Changes in the functional intensity of this circuit also involve synaptic remodeling [32]. It is therefore possible that in response to E_2_-treatment, LHN NTPDase3-IR hypocretin-orexin-containing neurons intensify their action on AN neuropeptide Y/Agouti-related protein-containing cells, thereby leading to increased NTPDase3 levels in both (medial and lateral) parts of the hypothalamus. If this was the case, one could speculate that the orexigenic effect of E_2 _may in some way involve the action of NTPDase3.

### Mitochondrial respiration measurements

It has been shown that interneuronal signaling is a highly ATP-dependent, energy-demanding process [33]. To supply the energy needs of neurotransmission, ATP is produced and maintained in neuronal mitochondria in a regulated fashion. We have previously proposed [34] that one potential mechanism down-regulating mitochondrial ATP production may involve uncoupling proteins (UCPs), specifically UCP2, which was only found in inhibitory neurons of the hypothalamus. However, the specific mechanism involved in the regulation of mitochondrial ATP levels in excitatory hypothalamic neurons is currently unknown. Therefore, the identification of NTPDase3 in mitochondria in synaptic or perikaryal sites of excitatory hypothalamic neurons might be the most novel and intriguing finding of this study, and warrants further experiments to elucidate the exact functional role of mitochondrial NTPDase3 in neurotransmission.

To confirm our morphological findings, we isolated synaptosomes from hypothalamic tissue homogenates and examined mitochondrial respiration in control versus suramin (an NTPDase inhibitor) treated samples. Suramin blockade of NTPDases reduced mitochondrial oxygen consumption in state 3 mitochondrial respiration by 30%, and also decreased the total mitochondrial respiratory capacity by 34%. These findings imply that induction of NTPDase activity in the mitochondria by hydrolyzing ATP to ADP increases mitochondrial state 3 respiration and the total mitochondrial respiration capacity. It should be noted that the polyoxometalate suramin is not a fully selective NTPDase3-inhibitor, as such inhibitors, to the best of our knowledge, for the 8 known NTPDases have not yet been found. However, suramin has been shown to be a potent inhibitor of NTPDase3 and other NTPDases [16], therefore the observed reduction in oxygen consumption in hypothalamic synaptosomes can be, at least in part, attributed to the inhibition of NTPDase3. This idea is supported by previous results on synaptosome fractions isolated from rat brain cortex and striatum [35], and from hippocampal synaptosomes [36]. These studies report a transient accumulation of ADP after addition of ATP followed by the subsequent metabolization of ADP to AMP and adenosine. As a result, the aforementioned studies argue against a considerable contribution by NTPDase1 and/or NTPDase2 and suggest that the observations would rather be compatible with a neuronal expression of NTPDase3.

Based on the present findings, it is tempting to speculate that an increase in the activity level of NTPDases (NTPDase3?) may result in the exhaustion of mitochondria (and the parent cell), whereas partial inhibition of NTPDases may be neuroprotective. Ongoing studies in our laboratory test this hypothesis. The reported pharmacological effects of polyoxometalates, such as suramin, seem to support this idea. For example, some data show that NTPDase inhibition is also antidiabetic [37], although the exact mechanisms through which the beneficial effects of NTPDase inhibitors act are unknown. Therefore, the present results rise the possibility that in the pancreas, inhibition of NTPDases (NTPDase3) may protect the insulin-producing beta cells from overt ATP consumption and consequential exhaustion.

## Conclusion

Altogether, the combined morphological and functional examination of neuronal NTPDase3-IR suggests a double cellular role for this enzyme in the hypothalamus: 1. as suggested earlier, the regulation of purinergic signaling via the production of specific ligands (ADP and AMP), and 2. the tuning of energy supply for (excitatory) neurotransmission by the hydrolysis of mitochondrial ATP.

## Competing interests

The authors declare that they have no competing interests.

## Authors' contributions

DSK was responsible for immunohistochemical studies including the "mirror" technique analysis; AZ and SD created the experimental design, supervised the experiments and wrote this report; KH and VLF carried out the mitochondrial respiration measurements and related analyses; AG and VS performed the western blot experiments; TB had a substantial share in overall data analysis, discussion of data, and participated in most of these experiments; DH and PS were responsible for the electron microscopic lab work and analyses. All authors read and approved the final manuscript.
